# Decoding the tumor microenvironment of triple-negative breast cancer: from immune evasion to precision immunotherapy

**DOI:** 10.3389/fimmu.2026.1887760

**Published:** 2026-08-25

**Authors:** Hongyu Cao, Meicheng Tao, Xuepeng Qin, Hong Guo

**Affiliations:** Department of Pharmacy, Shengjing Hospital of China Medical University, Shenyang, Liaoning, China

**Keywords:** checkpoint inhibitor combination, immune checkpoint blockade, immune evasion, macrophage, natural killer cell, neutrophil, triple-negative breast cancer, tumor-infiltrating lymphocytes

## Abstract

Triple-negative breast cancer (TNBC) represents a highly aggressive breast cancer subtype characterized by the absence of estrogen receptor, progesterone receptor, and HER2 expression, resulting in limited targeted therapeutic options and poor clinical outcomes. Although immune checkpoint inhibitors (ICIs) have transformed the treatment landscape of TNBC, therapeutic responses remain restricted to a subset of patients due to substantial heterogeneity within the tumor immune microenvironment (TIME). Increasing evidence indicates that immune evasion in TNBC is driven by complex interactions among tumor cells, immune populations, stromal components, and metabolic alterations. This review summarizes the dynamic landscape of the TNBC immune microenvironment, focusing on the functional roles of tumor-infiltrating lymphocytes, tumor-associated macrophages, tumor-associated neutrophils, natural killer cells, and myeloid-derived suppressor cells in regulating antitumor immunity and therapeutic resistance. We further discuss clinical advances of ICIs, including monotherapy and chemoimmunotherapy approaches, as well as emerging therapeutic strategies involving small molecules, bispecific antibodies, antibody–drug conjugates, and novel immune-based modalities.

## 1 Introduction

Triple-negative breast cancer (TNBC) accounts for approximately 15–20% of breast cancer cases and represents one of the most clinically aggressive molecular subtypes due to its high proliferative capacity, early metastatic potential, and limited availability of effective targeted therapies (1–3). Unlike hormone receptor-positive or HER2-positive breast cancers, TNBC lacks expression of estrogen receptor, progesterone receptor, and HER2, resulting in chemotherapy historically serving as the primary systemic treatment option (4). However, intrinsic and acquired chemotherapy resistance, together with frequent disease recurrence and metastasis, continues to contribute to unfavorable clinical outcomes (5, 6). Notably, TNBC exhibits greater immune infiltration and higher tumor mutational burden compared with other breast cancer subtypes, suggesting a unique biological context for immunotherapeutic intervention (7).

The tumor immune microenvironment (TIME) plays a fundamental role in determining TNBC progression, immune escape, and therapeutic response (8). Interactions between malignant cells and immune components, including cytotoxic T cells, regulatory T cells, macrophages, neutrophils, natural killer cells, and myeloid-derived suppressor cells, collectively shape an immunosuppressive niche that limits effective antitumor immunity (9). Although immune ICIs have achieved clinical success in selected TNBC populations, primary and acquired resistance remain major challenges (10). Therefore, a comprehensive understanding of immune regulation within the TNBC microenvironment is essential for developing rational combination strategies and advancing precision immunotherapy.

## 2 Tumor microenvironment in triple-negative breast cancer

### 2.1 Lymphocytic elements within the immune microenvironment

#### 2.1.1 CD8^+^ T cell subsets in triple-negative breast cancer

In TNBC, CD8^+^ T cells constitute the principal cytotoxic lymphocyte subset orchestrating antitumor surveillance (11). Following the recognition of tumor-associated antigens presented via MHC-I molecules, naive CD8^+^ T cells differentiate into effector cytotoxic T lymphocytes (CTLs). These CTLs eradicate malignant cells primarily through the exocytosis of perforin and granzymes, the secretion of IFN-γ, and the induction of target cell apoptosis (12, 13). Consistent with their antitumor function, increased CD8^+^ T-cell infiltration is associated with improved prognosis, prolonged survival, and enhanced therapeutic responses in TNBC (11, 14). However, the antitumor activity of CD8^+^ T cells is determined not only by their abundance but also by their functional state (15). Persistent antigen stimulation and immunosuppressive signals within the TNBC TME can induce CD8^+^ T-cell exhaustion, characterized by sustained expression of inhibitory receptors, including PD-1, TIM-3, and LAG-3, accompanied by reduced cytokine production and impaired cytotoxic capacity (16–18). CD8^+^ T-cell exhaustion represents a heterogeneous continuum rather than a terminal state (19). Progenitor exhausted/stem-like CD8^+^ T cells expressing TCF1 retain self-renewal capacity and proliferative potential, and these populations are considered critical responders to immune checkpoint blockade (20, 21). Conversely, terminally exhausted CD8^+^ T cells exhibit profound epigenetic scarring and display minimal functional rejuvenation following ICB therapy (22, 23). Furthermore, memory-like CD8^+^ T cell subsets contribute to durable immunological surveillance by conferring enhanced persistence and robust recall responses against recurrent tumor antigens (24–26).

#### 2.1.2 CD4^+^ helper t cells in triple-negative breast cancer

CD4^+^ helper T cells represent a heterogeneous population of adaptive immune cells that coordinate antitumor immune responses through functional specialization into distinct subsets, including Th1, Th2, Th17, and follicular helper T cells (Tfh) (27, 28). CD4^+^ T cells primarily regulate immune responses by modulating the activation and function of cytotoxic lymphocytes, antigen-presenting cells, B cells, and innate immune populations (29). In cancer immunity, CD4^+^ T-cell subsets critically influence the tumor microenvironment toward immune activation or immune tolerance (30–32). Among these subsets, Th1 cells are generally considered important mediators of antitumor immunity due to their production of IFN-γ, which enhances antigen presentation, promotes macrophage activation, and supports CD8^+^ T-cell-mediated tumor killing (33–35). Conversely, Th2 and Th17 responses may exhibit context-dependent effects in tumor progression (36). While Th17-derived cytokines can enhance immune recruitment under certain conditions, chronic IL-17 signaling may promote tumor-associated inflammation, angiogenesis, and immune suppression (37). Tfh cells, characterized by expression of CXCR5 and PD-1, regulate B-cell maturation and antibody production within tertiary lymphoid structures, which have been associated with improved immunotherapy responsiveness in several malignancies (38–40). In TNBC, CD4^+^ helper T cells contribute substantially to shaping the immune landscape and therapeutic response (41). Compared with hormone receptor-positive breast cancer subtypes, TNBC generally exhibits higher immune infiltration and greater enrichment of immune-related transcriptional signatures, suggesting increased immune interaction within the tumor microenvironment (42). However, the functional state of CD4^+^ T cells is highly dependent on local cytokine networks and tumor-derived suppressive signals. Persistent exposure to immunosuppressive factors, including TGF-β, IL-10, and metabolic stress signals, can induce CD4^+^ T-cell dysfunction and impair effective immune surveillance (43). Therefore, understanding the balance between immune-stimulatory and immune-regulatory CD4^+^ T-cell subsets is essential for developing rational immunotherapeutic strategies in TNBC.

#### 2.1.3 Regulatory T cells in triple-negative breast cancer

Tregs represent a specialized immunosuppressive CD4^+^ T-cell population that maintains peripheral immune tolerance under physiological conditions (44). However, within the tumor microenvironment, Tregs are frequently recruited and expanded by tumor-derived chemokines and inflammatory signals, where they become critical mediators of immune escape by actively suppressing antitumor immune responses (45, 46). In TNBC, Tregs are particularly enriched compared with other breast cancer subtypes, and increased infiltration of FOXP3^+^ Tregs has been detected in a substantial proportion of tumors (47). The accumulation of Tregs within TNBC lesions is associated with reduced immune activation, impaired effector T-cell function, and resistance to immune-based therapies (48, 49). Mechanistically, Tregs suppress antitumor immunity through multiple complementary pathways. Tregs secrete immunosuppressive cytokines, including IL-10 and TGF-β, which inhibit cytotoxic T lymphocyte activity, reduce inflammatory cytokine production, and promote the development of an immunosuppressive tumor microenvironment (44, 50). Tregs express high levels of immune checkpoint molecules, particularly CTLA-4, which competitively binds CD80/CD86 on antigen-presenting cells and reduces their costimulatory capacity, thereby limiting effective priming of tumor-reactive T cells (51). Furthermore, Treg-derived CTLA-4 signaling can induce indoleamine 2,3-dioxygenase (IDO) expression in dendritic cells, resulting in tryptophan depletion and accumulation of immunosuppressive metabolites that further inhibit effector T-cell proliferation and function (52). Tregs exert metabolic suppression through competition for essential growth factors, particularly IL-2. By constitutively expressing high levels of CD25, Tregs efficiently consume extracellular IL-2, depriving conventional CD4^+^ and CD8^+^ effector T cells of this critical survival and proliferation signal, thereby weakening antitumor immune responses (53). Tregs can directly suppress natural killer cells, dendritic cells, and macrophage activation through cell–cell interactions and inhibitory receptor signaling, further reinforcing immune tolerance within TNBC lesions (54, 55).

### 2.2 Tumor-associated macrophages in triple-negative breast cancer

TAMs are critical regulators of tumor progression, immune escape, and therapeutic resistance in TNBC (56). Depending on environmental signals, macrophages can adopt distinct functional states, including pro-inflammatory M1-like and immunosuppressive M2-like phenotypes (57, 58). In the TNBC TME, tumor-derived factors such as IL-10, TGF-β, and macrophage colony-stimulating factor (M-CSF) promote macrophage recruitment and polarization toward M2-like TAMs, thereby facilitating tumor growth and immune suppression (57, 59). TNBC exhibits increased infiltration of TAMs, particularly CD163^+^ and CD204^+^ macrophage populations. Elevated accumulation of these M2-associated TAMs is correlated with aggressive clinicopathological characteristics, including enhanced tumor proliferation, vascular invasion, poor differentiation, and unfavorable prognosis (58). TAMs contribute to TNBC progression by secreting immunosuppressive cytokines, including IL-10 and TGF-β, which impair CD8^+^ T-cell-mediated cytotoxicity, while also promoting angiogenesis, extracellular matrix remodeling, and metastatic dissemination (60, 61). TAMs have emerged as promising therapeutic targets. Inhibition of the CSF1R, a key regulator of macrophage survival and differentiation, can reduce TAM accumulation and enhance antitumor immunity (62, 63). However, TAM-targeting strategies alone often show limited efficacy, highlighting the need for combination approaches. For example, combined targeting of CSF1R and CXCR2 signaling has been shown to decrease TAMs while improving responses to PD-1 blockade (64). These findings emphasize the importance of targeting the broader immunosuppressive myeloid network and provide a rationale for integrating TAM-directed therapies with immune checkpoint inhibitors in TNBC.

### 2.3 Tumor-associated neutrophils in triple-negative breast cancer

Tumor-associated neutrophils (TANs) exhibit functional plasticity and can be broadly classified into antitumor N1 and tumor-promoting N2 phenotypes. The polarization process is strongly influenced by cytokine signaling within the TME (65). Pro-inflammatory signals, including IFN-β and other IFN-associated pathways, promote N1-like neutrophil activation characterized by enhanced inflammatory responses and tumoricidal activity (66). In contrast, tumor-derived TGF-β signaling drives TAN polarization toward an N2-like phenotype, which exhibits immunosuppressive functions and promotes tumor growth through secretion of VEGF-A, CXCL1, CXCL2, and matrix-remodeling factors (67–69). In TNBC, increased TAN infiltration and elevated neutrophil-to-lymphocyte ratios (NLRs) are frequently associated with poor prognosis and reduced therapeutic responses (70–72). TANs promote immune escape by suppressing cytotoxic lymphocyte activity, enhancing angiogenesis, and facilitating metastatic progression. Recruitment of neutrophils into tumor tissues is primarily regulated by C-X-C chemokine receptors, particularly CXCR1 and CXCR2, making these pathways attractive therapeutic targets (73, 74). The CXCR1/2 inhibitor reparixin has been evaluated in clinical studies involving metastatic breast cancer patients receiving chemotherapy (75, 76). Early-phase trials demonstrated acceptable safety profiles and clinical feasibility, although limited antitumor efficacy indicated that CXCR1/2 inhibition may require combination with other immunomodulatory strategies (77). Therefore, targeting TAN recruitment and polarization, together with immune checkpoint blockade or other TME-modulating approaches, may represent a promising strategy for overcoming immune resistance in TNBC.

### 2.4 NK cells and MDSCs in triple-negative breast cancer

Natural killer (NK) cells and myeloid-derived suppressor cells (MDSCs) represent two functionally distinct but highly interconnected immune populations that critically regulate antitumor immunity (78, 79). NK cells are essential components of innate immune surveillance and eliminate malignant cells through multiple mechanisms, including perforin/granzyme-mediated cytotoxicity, death receptor signaling, and antibody-dependent cellular cytotoxicity mediated by CD16/FcγRIII (80, 81). In TNBC, increased infiltration and activation of NK cells are generally associated with improved immune surveillance and favorable clinical outcomes (82, 83). However, the immunosuppressive TME, characterized by elevated levels of TGF-β, IL-10, hypoxia, and metabolic stress, can impair NK-cell cytotoxic function and promote an exhausted phenotype (84, 85). Therefore, restoring NK-cell activity through cytokine stimulation, metabolic modulation, or combination with immune checkpoint blockade represents a promising therapeutic strategy (86, 87). Conversely, MDSCs are major immunosuppressive myeloid populations that accumulate in TNBC and contribute to tumor progression and immune escape (88, 89). Based on phenotypic characteristics, MDSCs are broadly classified into monocytic (M-MDSCs) and polymorphonuclear/granulocytic (PMN-MDSCs) subsets (88, 90). These cells suppress antitumor immunity by inhibiting CD8^+^ T-cell and NK-cell function through mechanisms involving ARG1, inducible iNOS, ROS, and immunosuppressive cytokine production (91). Furthermore, MDSCs promote angiogenesis, metastasis, and resistance to immune checkpoint inhibitors by maintaining an immunosuppressive myeloid network within the TME (92, 93). Targeting MDSCs and restoring NK-cell activity have therefore emerged as potential approaches to enhance immunotherapy efficacy in TNBC. For example, inhibition of CSF1R and CXCR2 signaling has been shown to reduce suppressive myeloid populations, including TAMs and MDSCs, while improving responses to PD-1 blockade (94). In sum, the dynamic balance between cytotoxic NK-cell activity and MDSC-mediated immune suppression represents an important determinant of TNBC immune responsiveness and provides opportunities for developing rational combination immunotherapies (95, 96) (**Supplementary Figure 1**).

## 3 Clinical advances of immune checkpoint inhibitors in TNBC

### 3.1 PD-1/PD-L1 inhibitor

As a PD-1 inhibitor, pembrolizumab has demonstrated preliminary antitumor activity as a single-agent therapy in previously treated metastatic TNBC (97). However, its overall efficacy remains limited. Early clinical trials, including KEYNOTE-012 and KEYNOTE-086, reported objective response rates (ORRs) ranging approximately from 5% to 21%, indicating that although immune checkpoint blockade can provide durable clinical benefits for a subset of patients, the majority of individuals derive limited therapeutic benefit from monotherapy (98–100). Patients with PD-L1-positive tumors, particularly those with a combined positive score (CPS) ≥10, exhibited higher response rates and improved survival outcomes following pembrolizumab treatment, highlighting PD-L1 expression as an important predictive biomarker for immunotherapy responsiveness (101). Nevertheless, the clinical limitations of ICI monotherapy remain substantial, largely due to the highly heterogeneous immune landscape of TNBC (102, 103). Primary resistance may arise from multiple factors, including intrinsic tumor genomic alterations, insufficient infiltration of tumor-infiltrating lymphocytes (TILs), impaired antigen presentation, and activation of alternative immunosuppressive pathways (104–106). Notably, the anti-PD-L1/CTLA-4 bispecific antibody KN046 plus nab-paclitaxel for metastatic triple-negative breast cancer treatment (NCT03872791) (107). Therefore, although single-agent immunotherapy has introduced a novel therapeutic option for a proportion of TNBC patients, its restricted response rate and widespread resistance have driven extensive investigation into combination strategies aimed at enhancing antitumor immunity and overcoming the limitations of monotherapy.

### 3.2 Immunotherapy combined with chemotherapy

The combination of immune checkpoint inhibitors with chemotherapy has become a standard first-line therapeutic strategy for advanced TNBC, supported by evidence from several pivotal phase III clinical trials (108). The IMpassion130 trial was the first landmark study demonstrating that atezolizumab combined with nab-paclitaxel significantly prolonged overall survival in patients with PD-L1-positive metastatic TNBC, establishing a new paradigm for immune-based combination therapy in this disease setting (109). Subsequently, the KEYNOTE-355 trial further consolidated the role of chemoimmunotherapy by demonstrating that pembrolizumab plus chemotherapy (nab-paclitaxel, paclitaxel, carboplatin-based regimens) significantly improved survival in patients with metastatic TNBC harboring a CPS ≥10 (110). The mechanisms underlying the immunomodulatory effects of chemotherapy are multifaceted. A key mechanism involves the induction of immunogenic cell death, through which chemotherapeutic agents not only eliminate tumor cells but also promote the release of danger-associated molecular patterns (DAMPs), tumor antigens, and inflammatory mediators that stimulate adaptive immune responses (111). In addition, chemotherapy can reshape the immunosuppressive tumor microenvironment by reducing Tregs, MDSCs, and other inhibitory immune populations, thereby creating a more favorable context for ICI-mediated immune activation (112, 113). However, different chemotherapeutic agents exhibit distinct immunomodulatory properties, and the optimal selection of chemotherapy partners requires rigorous evaluation through high-quality clinical evidence to maximize therapeutic efficacy while minimizing toxicity (114).

## 4 Current advances in immunotherapeutic drugs for TNBC

### 4.1 Novel small-molecule drugs targeting the tumor immune microenvironment

Chemotherapy has long served as the cornerstone of systemic treatment for TNBC (115, 116). However, its clinical efficacy remains limited by the development of drug resistance and disease recurrence (117). In recent years, small-molecule agents and novel combination strategies capable of remodeling the TIME have been increasingly investigated for their ability to reverse immune tolerance and enhance antitumor responses (118, 119). Poly (ADP-ribose) polymerase (PARP) inhibitors, including olaparib and talazoparib, represent a major therapeutic advancement for patients with BRCA1/2-mutated TNBC (120, 121). Beyond their direct cytotoxic effects via synthetic lethality, PARP inhibitors trigger DNA damage accumulation that activates the cGAS–STING pathway, thereby amplifying type I interferon signaling, augmenting tumor immunogenicity, and facilitating immune cell recruitment (122, 123). These immunomodulatory properties provide a compelling mechanistic rationale for combining PARP inhibitors with ICIs to achieve synergistic antitumor activity (124). In addition to PARP inhibitors, targeted inhibitors of key oncogenic signaling cascades, such as the PI3K/AKT/mTOR and MAPK pathways, exhibit profound immunomodulatory effects in TNBC (125–127). Agents like the mTOR inhibitor everolimus and various MEK inhibitors can comprehensively reprogram the immunosuppressive TIME by altering intrinsic tumor cell signaling, modulating cytokine profiles, upregulating antigen presentation, altering PD-L1 expression, and sensitizing previously resistant malignant cells to immune-mediated cytotoxicity (128–130) (131, 132).

**Figure 1:**
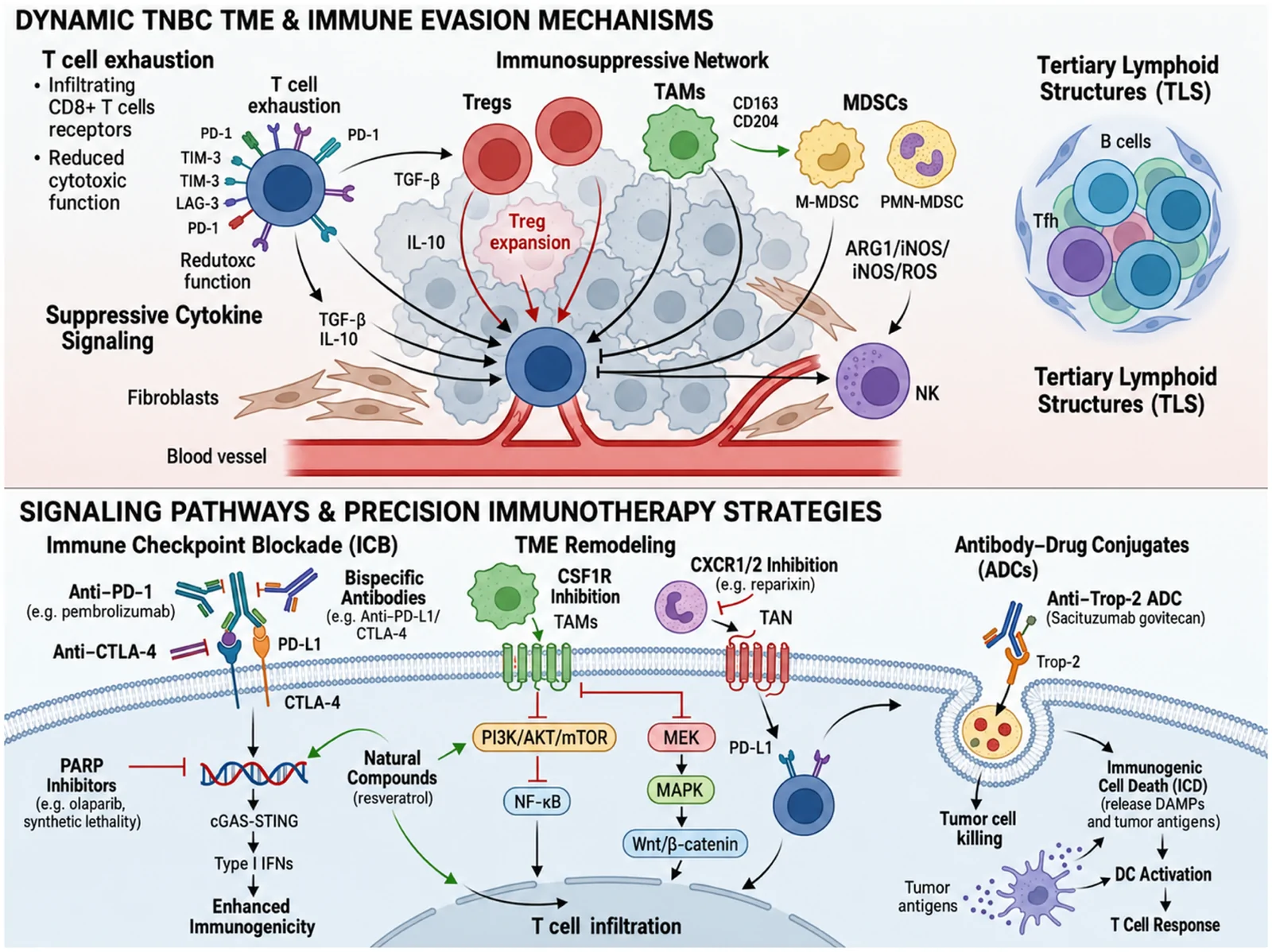
Signaling pathways and precision immunotherapy strategies for triple-negative breast cancer.

Concurrently, natural compounds have attracted increasing attention as potential immunomodulatory agents in TNBC (133). Bioactive phytochemicals, including curcumin, resveratrol, and triptolide, exert potent antitumor activities by regulating diverse signaling networks, such as autophagy, MAPK, and Wnt/β-catenin pathways (33, 134–136). These compounds inhibit TNBC cell proliferation, migration, and invasion, while potentially regulating immune-associated genes, inflammatory cytokine production, and immune cell function within the TME (137). Specifically, curcumin suppresses NF-κB-mediated inflammatory signaling to reduce immunosuppressive cytokine secretion (138, 139); resveratrol regulates oxidative stress-related pathways to bolster immune surveillance (140, 141); and triptolide impedes transcriptional programs driving tumor progression and immune evasion (33, 142, 143). Ultimately, the integration of these multi-targeted natural compounds into clinical regimens offers novel therapeutic avenues to enhance immune responsiveness and overcome immunotherapy resistance in TNBC (144) (**Figure 1**).

### 4.2 Bispecific antibodies and antibody–drug conjugates for TNBC

Antibody–drug conjugates (ADCs) represent another rapidly advancing therapeutic strategy that has substantially reshaped the treatment landscape of TNBC (145). By integrating the precise tumor-targeting specificity of monoclonal antibodies with the potent cytotoxicity of chemotherapeutic payloads, ADCs facilitate the selective intracellular delivery of anticancer agents (146). Specifically, sacituzumab govitecan (an anti-Trop-2 ADC) and trastuzumab deruxtecan (an anti-HER2 ADC effective in HER2-low breast cancer) have demonstrated robust clinical efficacy in advanced TNBC, providing critical therapeutic options beyond standard chemotherapy (147–149). Importantly, the therapeutic impact of ADCs extends beyond direct tumor cell eradication. ADC-induced immunogenic cell death (ICD) provokes the release of tumor neoantigens and DAMPs, which subsequently promote dendritic cell cross-presentation and amplify T-cell-mediated antitumor immunity (150, 151). This inherent immunomodulatory capacity provides a strong mechanistic rationale for combining ADCs with ICIs. Recent findings from the KEYNOTE-D19 trial revealed that combining sacituzumab govitecan with pembrolizumab significantly prolonged progression-free survival in patients with PD-L1-positive metastatic TNBC, providing important support for ADC–immunotherapy combinations in this setting (152).

## 5 Conclusion

Triple-negative breast cancer (TNBC) is a biologically and immunologically heterogeneous malignancy wherein therapeutic outcomes are largely dictated by intricate crosstalk between tumor cells and the surrounding immune microenvironment. Although immune checkpoint inhibitors (ICIs) have established a novel therapeutic paradigm for TNBC, durable clinical benefits remain confined to a subset of patients owing to multifaceted immune escape mechanisms, including T-cell exhaustion, enrichment of immunosuppressive populations, metabolic reprogramming, and stromal-mediated immune exclusion. Accumulating evidence demonstrates that tumor-infiltrating lymphocytes, regulatory T cells, tumor-associated macrophages, neutrophils, natural killer cells, and myeloid-derived suppressor cells collectively sculpt immune responsiveness and therapeutic resistance. Consequently, a comprehensive elucidation of the spatial and functional heterogeneity within the TNBC immune microenvironment is imperative for identifying actionable therapeutic vulnerabilities.

Future breakthroughs in TNBC immunotherapy will hinge on precision strategies that integrate high-resolution immune profiling, molecular characterization, and rational combinatorial regimens. Beyond conventional PD-1/PD-L1 blockade, emerging modalities—including immune microenvironment remodeling, antibody–drug conjugates, bispecific antibodies, small-molecule immunomodulators, and cellular immunotherapies—offer promising avenues to overcome resistance. Nevertheless, critical challenges persist regarding patient stratification, biomarker optimization, management of treatment-related toxicity, and the identification of synergistic therapeutic combinations. Incorporating single-cell sequencing, spatial transcriptomics, artificial intelligence-driven predictive models, and longitudinal immune monitoring will be indispensable for decoding dynamic immune states and guiding individualized immunotherapy. Ultimately, translating mechanistic insights into durable, clinically effective immune-based interventions remains essential to fundamentally improving patient outcomes in TNBC.

## Supplementary material

Supplementary Figure 1 Tumor microenvironment of triple-negative breast cancer.

## References

[1] Zagami P Carey LA . Triple negative breast cancer: Pitfalls and progress. NPJ Breast Cancer. (2022) 8:95. doi: 10.1038/s41523-022-00468-0 35987766 PMC9392735

[2] Yin L Duan JJ Bian XW Yu SC . Triple-negative breast cancer molecular subtyping and treatment progress. Breast Cancer Res. (2020) 22:61. doi: 10.1186/s13058-020-01296-5 32517735 PMC7285581

[3] Español A-J Sanchez Y Volpi S . Metronomic chemotherapy response in MDA-MB-231 triple-negative breast cancer cells under nicotine exposure. (2025) 49:1449–80.

[4] Obidiro O Battogtokh G Akala EO . Triple negative breast cancer treatment options and limitations: Future outlook. Pharmaceutics. (2023) 15:1796. doi: 10.20944/preprints202306.0074.v1 37513983 PMC10384267

[5] Xiong N Wu H Yu Z . Advancements and challenges in triple-negative breast cancer: A comprehensive review of therapeutic and diagnostic strategies. Front Oncol. (2024) 14:1405491. doi: 10.3389/fonc.2024.1405491 38863622 PMC11165151

[6] Raman R Debata S Govindarajan T Kumar P . Targeting triple-negative breast cancer: Resistance mechanisms and therapeutic advancements. Cancer Med. (2025) 14:e70803. doi: 10.1002/cam4.70803 40318146 PMC12048392

[7] Gao C Li H Liu C Xu X Zhuang J Zhou C . Tumor mutation burden and immune invasion characteristics in triple negative breast cancer: Genome high-throughput data analysis. Front Immunol. (2021) 12:650491. doi: 10.3389/fimmu.2021.650491 33968045 PMC8097167

[8] Guo Z Zhu Z Lin X Wang S Wen Y Wang L . Tumor microenvironment and immunotherapy for triple-negative breast cancer. biomark Res. (2024) 12:166. doi: 10.1186/s40364-024-00714-6 39741315 PMC11689763

[9] Niture S Ghosh S Jaboin J Seneviratne D . Tumor microenvironment dynamics of triple-negative breast cancer under radiation therapy. Int J Mol Sci. (2025) 26:2795. doi: 10.3390/ijms26062795 40141437 PMC11943269

[10] Page DB Pucilowska J Chun B Kim I Sanchez K Moxon N . A phase Ib trial of pembrolizumab plus paclitaxel or flat-dose capecitabine in 1st/2nd line metastatic triple-negative breast cancer. NPJ Breast Cancer. (2023) 9:53. doi: 10.1038/s41523-023-00541-2 37344474 PMC10284878

[11] Li X Gruosso T Zuo D Omeroglu A Meterissian S Guiot MC . Infiltration of CD8(+) T cells into tumor cell clusters in triple-negative breast cancer. Proc Natl Acad Sci USA. (2019) 116:3678–87. doi: 10.1073/pnas.1817652116 30733298 PMC6397588

[12] Raskov H Orhan A Christensen JP Gögenur I . Cytotoxic CD8(+) T cells in cancer and cancer immunotherapy. Br J Cancer. (2021) 124:359–67. doi: 10.1038/s41416-020-01048-4 32929195 PMC7853123

[13] Hay ZLZ Slansky JE . Granzymes: The molecular executors of immune-mediated cytotoxicity. Int J Mol Sci. (2022) 23:1833. doi: 10.3390/ijms23031833 35163755 PMC8836949

[14] Uenaka N Sato E Horimoto Y Kawai S Asaoka M Kaise H . CD8-positive T-cells are key immune cells for predicting the therapeutic effect of neoadjuvant chemotherapy in triple-negative breast cancer. Anticancer Res. (2024) 44:4525–36. doi: 10.21873/anticanres.17281 39348963

[15] Lee HK Ji HJ Shin SK Koo J Kim TH Kim CW . Targeting transforming growth factor-β2 by antisense oligodeoxynucleotide accelerates T cell-mediated tumor rejection in a humanized mouse model of triple-negative breast cancer. Cancer Immunol Immunother. (2022) 71:2213–26. doi: 10.1007/s00262-022-03157-w 35099588 PMC10992746

[16] Feng D Pu D Ren J Liu M Zhang Z Liu Z . CD8(+) T-cell exhaustion: Impediment to triple-negative breast cancer (TNBC) immunotherapy. Biochim Biophys Acta Rev Cancer. (2024) 1879:189193. doi: 10.1016/j.bbcan.2024.189193 39413858

[17] Chow A Perica K Klebanoff CA Wolchok JD . Clinical implications of T cell exhaustion for cancer immunotherapy. Nat Rev Clin Oncol. (2022) 19:775–90. doi: 10.1038/s41571-022-00689-z 36216928 PMC10984554

[18] Fu T Jin X He M Chen YY Yang YS Chen L . Interferon-induced senescent CD8(+) T cells reduce anti-PD1 immunotherapy efficacy in early triple-negative breast cancer. Sci Transl Med. (2025) 17:eadj7808. doi: 10.1126/scitranslmed.adj7808 40929245

[19] Xie H Xi X Lei T Liu H Xia Z . CD8(+) T cell exhaustion in the tumor microenvironment of breast cancer. Front Immunol. (2024) 15:1507283. doi: 10.3389/fimmu.2024.1507283 39717767 PMC11663851

[20] Lee J Lee K Bae H Lee K Lee S Ma J . IL-15 promotes self-renewal of progenitor exhausted CD8 T cells during persistent antigenic stimulation. Front Immunol. (2023) 14:1117092. doi: 10.3389/fimmu.2023.1117092 37409128 PMC10319055

[21] Gill AL Wang PH Lee J Hudson WH Ando S Araki K . PD-1 blockade increases the self-renewal of stem-like CD8 T cells to compensate for their accelerated differentiation into effectors. Sci Immunol. (2023) 8:eadg0539. doi: 10.1126/sciimmunol.adg0539 37624909 PMC10798572

[22] Miller BC Sen DR Al Abosy R Bi K Virkud YV LaFleur MW . Subsets of exhausted CD8(+) T cells differentially mediate tumor control and respond to checkpoint blockade. Nat Immunol. (2019) 20:326–36. doi: 10.1038/s41590-019-0312-6 30778252 PMC6673650

[23] Chen M Fu Z Wu C . Tumor-derived exosomal ICAM1 promotes bone metastasis of triple-negative breast cancer by inducing CD8+ T cell exhaustion. Int J Biochem Cell Biol. (2024) 175:106637. doi: 10.1016/j.biocel.2024.106637 39147124

[24] Rahim MK Okholm TLH Jones KB McCarthy EE Liu CC Yee JL . Dynamic CD8(+) T cell responses to cancer immunotherapy in human regional lymph nodes are disrupted in metastatic lymph nodes. Cell. (2023) 186:1127–1143.e1118. doi: 10.1016/j.cell.2023.02.021 36931243 PMC10348701

[25] Virassamy B Caramia F Savas P Sant S Wang J Christo SN . Intratumoral CD8(+) T cells with a tissue-resident memory phenotype mediate local immunity and immune checkpoint responses in breast cancer. Cancer Cell. (2023) 41:585–601.e588. doi: 10.1016/j.ccell.2023.01.004 36827978

[26] Zhao Z Ma X Cai Z . The potential role of CD8+ cytotoxic T lymphocytes and one branch connected with tissue-resident memory in non-luminal breast cancer. PeerJ. (2024) 12:e17667. doi: 10.7717/peerj.17667 39006029 PMC11246025

[27] Zhang H Qin G Yu H Han X Zhu S . Comprehensive genomic and immunophenotypic analysis of CD4 T cell infiltrating human triple-negative breast cancer. Cancer Immunol Immunother. (2021) 70:1649–65. doi: 10.1007/s00262-020-02807-1 33301062 PMC8139937

[28] Hong CC Yao S McCann SE Dolnick RY Wallace PK Gong Z . Pretreatment levels of circulating Th1 and Th2 cytokines, and their ratios, are associated with ER-negative and triple negative breast cancers. Breast Cancer Res Treat. (2013) 139:477–88. doi: 10.1007/s10549-013-2549-3 23624818 PMC3912696

[29] Accogli T Bruchard M Végran F . Modulation of CD4 T cell response according to tumor cytokine microenvironment. Cancers (Basel). (2021) 13:373. doi: 10.3390/cancers13030373 33498483 PMC7864169

[30] Leon-Ferre RA Jonas SF Salgado R Loi S de Jong V Carter JM . Tumor-infiltrating lymphocytes in triple-negative breast cancer. Jama. (2024) 331:1135–44. doi: 10.1001/jama.2024.3056 38563834 PMC10988354

[31] Chandraganti S Sams C Sahoo S Feng B O'Connell I Li L . CD4+ T cells sensitize quasimesenchymal breast tumors lacking CD73 to anti-CTLA4 immune checkpoint blockade therapy. Cancer Res Commun. (2026) 6:1278–94. doi: 10.1158/2767-9764.crc-26-0304 42118667 PMC13227059

[32] Okuuchi A Ibuki Y Katsuki S Minami K Tatekawa S Tamari K . Hyperthermia combined with anti-CTLA-4 antibody induces tumor microenvironment remodeling involving CD4(+) T cells in local and distant antitumor effects in a murine triple-negative breast cancer. Cancers (Basel). (2026) 18:1295. doi: 10.3390/cancers18081295 42073618 PMC13114660

[33] Cai J Zhong M Xu J Cheng H Xu S . Codelivery of triptolide and IFN-γ to boost antitumor immunity for triple-negative breast cancer. Int Immunopharmacol. (2023) 120:110346. doi: 10.1016/j.intimp.2023.110346 37210915

[34] Cecil DL Herendeen D Slota M O'Meara MM Dang Y Corulli L . Identification and validation of Th1-selective epitopes derived from proteins overexpressed in breast cancer stem cells. Vaccines (Basel). (2025) 13:525. doi: 10.3390/vaccines13050525 40432134 PMC12115844

[35] Anwar A Lepore C Czerniecki BJ Koski GK Showalter LE . PIM kinase inhibitor AZD1208 in conjunction with Th1 cytokines potentiate death of breast cancer cells*in vitro*while also maximizing suppression of tumor growth*in vivo* when combined with immunotherapy. Cell Immunol. (2024) 397-398:104805. doi: 10.1016/j.cellimm.2024.104805 38244265

[36] Guo W Tan J Wang L Egelston CA Simons DL Ochoa A . Tumor draining lymph nodes connected to cold triple-negative breast cancers are characterized by Th2-associated microenvironment. Nat Commun. (2024) 15:8592. doi: 10.1038/s41467-024-52577-y 39366933 PMC11452381

[37] Faucheux L Grandclaudon M Perrot-Dockès M Sirven P Berger F Hamy AS . A multivariate Th17 metagene for prognostic stratification in T cell non-inflamed triple negative breast cancer. Oncoimmunology. (2019) 8:e1624130. doi: 10.1080/2162402x.2019.1624130 31428522 PMC6685521

[38] Noël G Fontsa ML Garaud S De Silva P de Wind A Van den Eynden GG . Functional Th1-oriented T follicular helper cells that infiltrate human breast cancer promote effective adaptive immunity. J Clin Invest. (2021) 131:e139905. doi: 10.1172/JCI139905 34411002 PMC8483751

[39] Bronsert P von Schoenfeld A Villacorta Hidalgo J Kraft S Pfeiffer J Erbes T . High numbers and densities of PD1(+) T-follicular helper cells in triple-negative breast cancer draining lymph nodes are associated with lower survival. Int J Mol Sci. (2020) 21:5948. doi: 10.3390/ijms21175948 32824917 PMC7504397

[40] Garaud S Dieu-Nosjean MC Willard-Gallo K . T follicular helper and B cell crosstalk in tertiary lymphoid structures and cancer immunotherapy. Nat Commun. (2022) 13:2259. doi: 10.1038/s41467-022-29753-z 35473931 PMC9043192

[41] Rosemblit C Datta J Lowenfeld L Xu S Basu A Kodumudi K . Oncodriver inhibition and CD4(+) Th1 cytokines cooperate through Stat1 activation to induce tumor senescence and apoptosis in HER2+ and triple negative breast cancer: Implications for combining immune and targeted therapies. Oncotarget. (2018) 9:23058–77. doi: 10.18632/oncotarget.25208 29796172 PMC5955413

[42] Liu C Li Y Xing X Zhuang J Wang J Wang C . Immunogenomic landscape analyses of immune molecule signature-based risk panel for patients with triple-negative breast cancer. Mol Ther Nucleic Acids. (2022) 28:670–84. doi: 10.1016/j.omtn.2022.04.034 35614988 PMC9123090

[43] Furukawa N Stearns V Santa-Maria CA Popel AS . The tumor microenvironment and triple-negative breast cancer aggressiveness: Shedding light on mechanisms and targeting. Expert Opin Ther Targets. (2022) 26:1041–56. doi: 10.1080/14728222.2022.2170779 36657483 PMC10189896

[44] Malla RR Vasudevaraju P Vempati RK Rakshmitha M Merchant N Nagaraju GP . Regulatory T cells: Their role in triple-negative breast cancer progression and metastasis. Cancer. (2022) 128:1171–83. doi: 10.1002/cncr.34084 34990009

[45] Zahran AM El-Badawy O Kamel LM Rayan A Rezk K Abdel-Rahim MH . Accumulation of regulatory T cells in triple negative breast cancer can boost immune disruption. Cancer Manag Res. (2021) 13:6019–29. doi: 10.2147/cmar.s285128 34377021 PMC8349183

[46] Xydia M Rahbari R Ruggiero E Macaulay I Tarabichi M Lohmayer R . Common clonal origin of conventional T cells and induced regulatory T cells in breast cancer patients. Nat Commun. (2021) 12:1119. doi: 10.1038/s41467-021-21297-y 33602930 PMC7893042

[47] Goda N Nakashima C Nagamine I Otagaki S . The effect of intratumoral interrelation among FOXP3+ regulatory T cells on treatment response and survival in triple-negative breast cancer. Cancers (Basel). (2022) 14:2138. doi: 10.3390/cancers14092138 35565267 PMC9104991

[48] Fattori S Le Roy A Houacine J Robert L Abes R Gorvel L . CD25high effector regulatory T cells hamper responses to PD-1 blockade in triple-negative breast cancer. Cancer Res. (2023) 83:3026–44. doi: 10.1158/0008-5472.can-23-0613 37379438 PMC10502453

[49] Huang P Zhou X Zheng M Yu Y Jin G Zhang S . Regulatory T cells are associated with the tumor immune microenvironment and immunotherapy response in triple-negative breast cancer. Front Immunol. (2023) 14:1263537. doi: 10.3389/fimmu.2023.1263537 37767092 PMC10521732

[50] Scott EN Gocher AM Workman CJ Vignali DAA . Regulatory T cells: Barriers of immune infiltration into the tumor microenvironment. Front Immunol. (2021) 12:702726. doi: 10.3389/fimmu.2021.702726 34177968 PMC8222776

[51] Sobhani N Tardiel-Cyril DR Davtyan A Generali D Roudi R Li Y . CTLA-4 in regulatory T cells for cancer immunotherapy. Cancers (Basel). (2021) 13:1440. doi: 10.3390/cancers13061440 33809974 PMC8005092

[52] Li P Wu R Li K Yuan W Zeng C Zhang Y . IDO inhibition facilitates antitumor immunity of Vγ9Vδ2 T cells in triple-negative breast cancer. Front Oncol. (2021) 11:679517. doi: 10.3389/fonc.2021.679517 34381711 PMC8351331

[53] Yofe I Landsberger T Yalin A Solomon I Costoya C Demane DF . Anti-CTLA-4 antibodies drive myeloid activation and reprogram the tumor microenvironment through FcγR engagement and type I interferon signaling. Nat Cancer. (2022) 3:1336–50. doi: 10.1038/s43018-022-00447-1 36302895

[54] You Y Wei L Dai M Zhao G Fan K Dang T . BMP9 potentiates immunotherapy in triple-negative breast cancer by suppressing Tregs infiltration via the PRKDC-CCL2 axis. Cancer Lett. (2026) 639:218175. doi: 10.1016/j.canlet.2025.218175 41360160

[55] Manukian G Kivolowitz C DeAngelis T Shastri AA Savage JE Camphausen K . Caloric restriction impairs regulatory T cells within the tumor microenvironment after radiation and primes effector T cells. Int J Radiat Oncol Biol Phys. (2021) 110:1341–9. doi: 10.1016/j.ijrobp.2021.02.029 33647370 PMC8286289

[56] Xia Q Chen X Ma Q Wen X . M2 macrophages predicted the prognosis of breast cancer by combing a novel immune cell signature and promoted cell migration and invasion of cancer cells *in vitro*. (2024) 48:217–28. doi: 10.32604/biocell.2023.027414

[57] Pe KCS Saetung R Yodsurang V Chaotham C Suppipat K Chanvorachote P . Triple-negative breast cancer influences a mixed M1/M2 macrophage phenotype associated with tumor aggressiveness. PloS One. (2022) 17:e0273044. doi: 10.1371/journal.pone.0273044 35960749 PMC9374254

[58] Qiu X Zhao T Luo R Qiu R Li Z . Tumor-associated macrophages: key players in triple-negative breast cancer. Front Oncol. (2022) 12:772615. doi: 10.3389/fonc.2022.772615 35237507 PMC8882594

[59] Li Y Han X Lin Z Wang C Fu Z Sun Q . G6PD activation in TNBC cells induces macrophage recruitment and M2 polarization to promote tumor progression. Cell Mol Life Sci. (2023) 80:165. doi: 10.1007/s00018-023-04810-y 37237244 PMC11073185

[60] Hirano R Okamoto K Shinke M Sato M Watanabe S Watanabe H . Tissue-resident macrophages are major tumor-associated macrophage resources, contributing to early TNBC development, recurrence, and metastases. Commun Biol. (2023) 6:144. doi: 10.1038/s42003-023-04525-7 36737474 PMC9898263

[61] Cheng N Bei Y Song Y Zhang W Xu L Zhang W . B7-H3 augments the pro-angiogenic function of tumor-associated macrophages and acts as a novel adjuvant target for triple-negative breast cancer therapy. Biochem Pharmacol. (2021) 183:114298. doi: 10.1016/j.bcp.2020.114298 33153969

[62] Pedroza DA Yuan X Liu F Chan HL Zhang C Bowie W . Anti-CSF-1R therapy with combined immuno-chemotherapy coordinate an adaptive immune response to eliminate macrophage enriched triple negative breast cancers. Nat Commun. (2026) 17:1193. doi: 10.1038/s41467-025-67964-2 41484081 PMC12858953

[63] Singh S Lee N Pedroza DA Bado IL Hamor C Zhang L . Chemotherapy coupled to macrophage inhibition induces T-cell and B-cell infiltration and durable regression in triple-negative breast cancer. Cancer Res. (2022) 82:2281–97. doi: 10.1158/0008-5472.can-21-3714 35442423 PMC9219596

[64] Poissonnier A Rugo HS Horton W Kim T DeLuca A Egeland EV . CSF1R inhibition with chemotherapy relieves systemic immune suppression in patients with metastatic triple-negative breast cancer and boosts anti-PD-1 efficacy in transgenic mammary tumors. Clin Cancer Res. (2026) 32:2832–49. doi: 10.1158/1078-0432.ccr-25-4236 41894563 PMC13376889

[65] Obeagu EI Ezeala CC . Neutrophils as key regulators of tumor microenvironment in breast cancer: a focus on N1 and N2 polarization. Ann Med Surg (Lond). (2025) 87:3509–22. doi: 10.1097/ms9.0000000000003269 40486595 PMC12140695

[66] Ralph SJ Reynolds MJ . Intratumoral pro-oxidants promote cancer immunotherapy by recruiting and reprogramming neutrophils to eliminate tumors. Cancer Immunol Immunother. (2023) 72:527–42. doi: 10.1007/s00262-022-03248-8 36066649 PMC9446783

[67] Zheng C Xu X Wu M Xue L Zhu J Xia H . Neutrophils in triple-negative breast cancer: an underestimated player with increasingly recognized importance. Breast Cancer Res. (2023) 25:88. doi: 10.1186/s13058-023-01676-7 37496019 PMC10373263

[68] Wen S Feng T Fan Y . Tumor-associated neutrophils in breast cancer: an angel or a devil? (2025) 16:2025. doi: 10.3389/fimmu.2025.1593156 PMC12187804 40568594

[69] Zhou Y Shen G Zhou X Li J . Therapeutic potential of tumor-associated neutrophils: dual role and phenotypic plasticity. Signal Transduction Targeted Ther. (2025) 10:178. doi: 10.1038/s41392-025-02242-7 40461514 PMC12134342

[70] Cupp MA Cariolou M Tzoulaki I Aune D Evangelou E Berlanga-Taylor AJ . Neutrophil to lymphocyte ratio and cancer prognosis: an umbrella review of systematic reviews and meta-analyses of observational studies. BMC Med. (2020) 18:360. doi: 10.1186/s12916-020-01817-1 33213430 PMC7678319

[71] García-Escobar A Vera-Vera S Tébar-Márquez D Rivero-Santana B Jurado-Román A Jiménez-Valero S . Neutrophil-to-lymphocyte ratio an inflammatory biomarker, and prognostic marker in heart failure, cardiovascular disease and chronic inflammatory diseases: new insights for a potential predictor of anti-cytokine therapy responsiveness. Microvasc Res. (2023) 150:104598. doi: 10.1016/j.mvr.2023.104598 37633337

[72] Gao S Tang W Zuo B Mulvihill L Yu J Yu Y . The predictive value of neutrophil-to-lymphocyte ratio for overall survival and pathological complete response in breast cancer patients receiving neoadjuvant chemotherapy. Front Oncol. (2022) 12:1065606. doi: 10.3389/fonc.2022.1065606 36727046 PMC9885149

[73] Schott AF Goldstein LJ Cristofanilli M Ruffini PA McCanna S Reuben JM . Phase Ib pilot study to evaluate reparixin in combination with weekly paclitaxel in patients with HER-2-negative metastatic breast cancer. Clin Cancer Res. (2017) 23:5358–65. doi: 10.1158/1078-0432.ccr-16-2748 28539464 PMC5600824

[74] SenGupta S Hein LE Xu Y Zhang J Konwerski JR Li Y . Triple-negative breast cancer cells recruit neutrophils by secreting TGF-β and CXCR2 ligands. Front Immunol. (2021) 12:659996. doi: 10.3389/fimmu.2021.659996 33912188 PMC8071875

[75] Goldstein LJ Perez RP Yardley D Han LK Reuben JM Gao H . A window-of-opportunity trial of the CXCR1/2 inhibitor reparixin in operable HER-2-negative breast cancer. Breast Cancer Res. (2020) 22:4. doi: 10.1186/s13058-019-1243-8 31924241 PMC6954543

[76] Goldstein LJ Mansutti M Levy C Chang JC Henry S Fernandez-Perez I . A randomized, placebo-controlled phase 2 study of paclitaxel in combination with reparixin compared to paclitaxel alone as front-line therapy for metastatic triple-negative breast cancer (fRida). Breast Cancer Res Treat. (2021) 190:265–75. doi: 10.1007/s10549-021-06367-5 34476645 PMC8558154

[77] Ha H Debnath B Neamati N . Role of the CXCL8-CXCR1/2 axis in cancer and inflammatory diseases. Theranostics. (2017) 7:1543–88. doi: 10.7150/thno.15625 28529637 PMC5436513

[78] Thacker G Henry S Nandi A Debnath R Singh S Nayak A . Immature natural killer cells promote progression of triple-negative breast cancer. Sci Transl Med. (2023) 15:eabl4414. doi: 10.1126/scitranslmed.abl4414 36888695 PMC10875969

[79] Park JD Kim KS Choi SH Jo GH Choi JH Park SW . ELK3 modulates the antitumor efficacy of natural killer cells against triple negative breast cancer by regulating mitochondrial dynamics. J Immunother Cancer. (2022) 10:e004825. doi: 10.1136/jitc-2022-004825 35858708 PMC9305827

[80] Chen M Zhang B Mu X Zhang B Yang T Zhang G . Recent advances in tumor immunotherapy based on NK cells. (2025) 16:2025. doi: 10.3389/fimmu.2025.1595533 PMC12367655 40852706

[81] Maia A Tarannum M Lérias JR Piccinelli S Borrego LM Maeurer M . Building a better defense: expanding and improving natural killer cells for adoptive cell therapy. Cells. (2024) 13:451. doi: 10.3390/cells13050451 38474415 PMC10930942

[82] Alfahdawi AJ Mohammed Sameen A Mohammed Hussien A Al-Bayatee NT Hafdi Abdtawfeeq T Rashied RM . CTL-NK signature based on immune gene profiling and IHC predicts clinical outcomes in triple-negative breast cancer. Cancer Invest. (2026) 44:567–84. doi: 10.1080/07357907.2026.2644899 41883034

[83] Liu Z Ding M Qiu P Pan K Guo Q . Natural killer cell-related prognostic risk model predicts prognosis and treatment outcomes in triple-negative breast cancer. Front Immunol. (2023) 14:1200282. doi: 10.3389/fimmu.2023.1200282 37520534 PMC10373504

[84] Kennedy PR Arvindam US Phung SK Ettestad B Feng X Li Y . Metabolic programs drive function of therapeutic NK cells in hypoxic tumor environments. Sci Adv. (2024) 10:eadn1849. doi: 10.17504/protocols.io.5qpvo3y9xv4o/v1 39475618 PMC11524192

[85] Ma S Zhao Y Lee WC Ong LT Lee PL Jiang Z . Hypoxia induces HIF1α-dependent epigenetic vulnerability in triple negative breast cancer to confer immune effector dysfunction and resistance to anti-PD-1 immunotherapy. Nat Commun. (2022) 13:4118. doi: 10.1038/s41467-022-31764-9 35840558 PMC9287350

[86] Wang Y Jiang Y Ding F Lu J Huang T Zhong G . Phenotypes and cytokines of NK cells in triple-negative breast cancer resistant to checkpoint blockade immunotherapy. Breast Cancer Res. (2025) 27:51. doi: 10.1186/s13058-025-02003-y 40181426 PMC11969778

[87] Palakurthi B Fross SR Guldner IH Aleksandrovic E Liu X Martino AK . Targeting CXCL16 and STAT1 augments immune checkpoint blockade therapy in triple-negative breast cancer. Nat Commun. (2023) 14:2109. doi: 10.1038/s41467-023-37727-y 37055410 PMC10101955

[88] Kajihara N Kobayashi T Otsuka R Nio-Kobayashi J Oshino T Takahashi M . Tumor-derived interleukin-34 creates an immunosuppressive and chemoresistant tumor microenvironment by modulating myeloid-derived suppressor cells in triple-negative breast cancer. Cancer Immunol Immunother. (2023) 72:851–64. doi: 10.1007/s00262-022-03293-3 36104597 PMC10992550

[89] Han J Wan M Ma Z Yi H . Regulation of DNA-PK activity promotes the progression of TNBC via enhancing the immunosuppressive function of myeloid-derived suppressor cells. Cancer Med. (2023) 12:5939–52. doi: 10.1002/cam4.5387 PMC10028116 36373232

[90] Gong B Zheng L . Subtype-specific heterogeneity of myeloid-derived suppressor cells in breast cancer: current insights and future directions. PeerJ. (2026) 14:e20937. doi: 10.7717/peerj.20937 42004698 PMC13086029

[91] Wu Y Yi M Niu M Mei Q Wu K . Myeloid-derived suppressor cells: an emerging target for anticancer immunotherapy. Mol Cancer. (2022) 21:184. doi: 10.1186/s12943-022-01657-y 36163047 PMC9513992

[92] Yang Y Liu Z Kaysar P Han Y Ni B Li L . Delta-like ligand 4 mediated myeloid-derived suppressor cell metabolic reprogramming promotes neoadjuvant therapy resistance in titin-inactivated triple-negative breast cancer. Mol BioMed. (2025) 6:128. doi: 10.1186/s43556-025-00372-6 41326912 PMC12669439

[93] Zhao H Tian H Nuerbieke T Ma K Li W Niu X . Immunotherapy resistance in triple-negative breast cancer: mechanisms and emerging therapeutic strategies. Cancer Lett. (2026) 658:218690. doi: 10.1016/j.canlet.2026.218690 42323003

[94] Wang Q Li S Wu Y Yu X Dai Y Wang Y . Gene signature-based drug screening reveals ponatinib enhances immunotherapy efficacy in triple-negative breast cancer by reversing MDSC-mediated immunosuppressive tumor microenvironment. Res (Wash D C). (2025) 8:915. doi: 10.34133/research.0915 41079672 PMC12508528

[95] Zalfa C Paust S . Natural killer cell interactions with myeloid derived suppressor cells in the tumor microenvironment and implications for cancer immunotherapy. Front Immunol. (2021) 12:633205. doi: 10.3389/fimmu.2021.633205 34025641 PMC8133367

[96] Fabian KP Padget MR Donahue RN Solocinski K Robbins Y Allen CT . PD-L1 targeting high-affinity NK (t-haNK) cells induce direct antitumor effects and target suppressive MDSC populations. J Immunother Cancer. (2020) 8:e000450. doi: 10.1136/jitc-2019-000450 32439799 PMC7247398

[97] Cortes J Lipatov O Im SA Goncalves A Lee KS Schmid P . Association of potential biomarkers with clinical outcomes in metastatic triple-negative breast cancer treated with pembrolizumab or chemotherapy. NPJ Breast Cancer. (2025) 11:109. doi: 10.1038/s41523-025-00814-y 41038856 PMC12491503

[98] Wu S Ge A Deng X Liu L Wang Y . Evolving immunotherapeutic solutions for triple-negative breast carcinoma. Cancer Treat Rev. (2024) 130:102817. doi: 10.1016/j.ctrv.2024.102817 39154410

[99] Nanda R Chow LQ Dees EC Berger R Gupta S Geva R . Pembrolizumab in patients with advanced triple-negative breast cancer: phase Ib KEYNOTE-012 study. J Clin Oncol. (2016) 34:2460–7. doi: 10.1200/jco.2015.64.8931 27138582 PMC6816000

[100] Buisseret L Bareche Y Venet D Girard E Gombos A Emonts P . The long and winding road to biomarkers for immunotherapy: a retrospective analysis of samples from patients with triple-negative breast cancer treated with pembrolizumab. ESMO Open. (2024) 9:102964. doi: 10.1016/j.esmoop.2024.102964 38703428 PMC11087916

[101] Ilieva N Pencheva M Hadzhiev H Tashkova D Daskalova E Georgiev P . Impact of neoadjuvant therapy on PD-L1 expression in triple-negative breast cancer and correlation with clinicopathological factors. Diagnostics (Basel). (2024) 14:2672. doi: 10.3390/diagnostics14232672 39682581 PMC11639797

[102] Hammerl D Martens JWM Timmermans M Smid M Trapman-Jansen AM Foekens R . Spatial immunophenotypes predict response to anti-PD1 treatment and capture distinct paths of T cell evasion in triple negative breast cancer. Nat Commun. (2021) 12:5668. doi: 10.1038/s41467-021-25962-0 34580291 PMC8476574

[103] Griguolo G Tosi A Dieci MV Fineberg S Rossi V Ventura A . A comprehensive profiling of the immune microenvironment of breast cancer brain metastases. Neuro Oncol. (2022) 24:2146–58. doi: 10.1093/neuonc/noac136 35609559 PMC9713504

[104] Banerjee R Maitra I Bhattacharya T Banerjee M Ramanathan G Rayala SK . Next-generation biomarkers for prognostic and potential therapeutic enhancement in triple negative breast cancer. Crit Rev Oncol Hematol. (2024) 201:104417. doi: 10.1016/j.critrevonc.2024.104417 38901639

[105] Qian XL Xia XQ Li YQ Jia YM Sun YY Song YM . Effects of tumor-infiltrating lymphocytes on nonresponse rate of neoadjuvant chemotherapy in patients with invasive breast cancer. Sci Rep. (2023) 13:9256. doi: 10.1038/s41598-023-36517-2 37286786 PMC10247751

[106] Kim H Choi JM Lee KM . Immune checkpoint blockades in triple-negative breast cancer: Current state and molecular mechanisms of resistance. Biomedicines. (2022) 10:1130. doi: 10.3390/biomedicines10051130 35625867 PMC9138553

[107] Li Q Liu J Zhang Q Ouyang Q Zhang Y Liu Q . The anti-PD-L1/CTLA-4 bispecific antibody KN046 in combination with nab-paclitaxel in first-line treatment of metastatic triple-negative breast cancer: a multicenter phase II trial. Nat Commun. (2024) 15:1015. doi: 10.1038/s41467-024-45160-y 38310192 PMC10838317

[108] Emens LA Adams S Barrios CH Diéras V Iwata H Loi S . First-line atezolizumab plus nab-paclitaxel for unresectable, locally advanced, or metastatic triple-negative breast cancer: IMpassion130 final overall survival analysis. Ann Oncol. (2021) 32:983–93. doi: 10.1016/j.annonc.2021.05.355 34272041

[109] Stimes N Stanbery L Albrethsen M Trivedi C Hamouda D Dworkin L . Small-cell breast carcinoma with response to atezolizumab: a case report. Immunotherapy. (2022). doi: 10.2217/imt-2021-0100 35481350

[110] Cortes J Cescon DW Rugo HS Nowecki Z Im SA Yusof MM . Pembrolizumab plus chemotherapy versus placebo plus chemotherapy for previously untreated locally recurrent inoperable or metastatic triple-negative breast cancer (KEYNOTE-355): a randomised, placebo-controlled, double-blind, phase 3 clinical trial. Lancet. (2020) 396:1817–28. doi: 10.1016/s0140-6736(20)32531-9 33278935

[111] Chen Z Zhang Q Huang Q Liu Z Zeng L Zhang L . Photothermal MnO(2) nanoparticles boost chemo-photothermal therapy-induced immunogenic cell death in tumor immunotherapy. Int J Pharm. (2022) 617:121578. doi: 10.1016/j.ijpharm.2022.121578 35176333

[112] Koh SB Ellisen LW . Immune activation and evolution through chemotherapy plus checkpoint blockade in triple-negative breast cancer. Cancer Cell. (2021) 39:1562–4. doi: 10.1016/j.ccell.2021.11.001 34767761

[113] Carpen L Falvo P Orecchioni S Mitola G Hillje R Mazzara S . A single-cell transcriptomic landscape of innate and adaptive intratumoral immunity in triple negative breast cancer during chemo- and immunotherapies. Cell Death Discov. (2022) 8:106. doi: 10.1038/s41420-022-00893-x 35260564 PMC8904804

[114] Bianchini G De Angelis C Licata L Gianni L . Treatment landscape of triple-negative breast cancer - expanded options, evolving needs. Nat Rev Clin Oncol. (2022) 19:91–113. doi: 10.1038/s41571-021-00565-2 34754128

[115] Hong C-E Lyu S-Y . VCA augments doxorubicin efficacy in triple-negative breast cancer: Evidence for multi-pathway synergism. (2025) 49:2377–97. doi: 10.32604/biocell.2025.072360

[116] Ma L Jiang Z Hou X Xu Y Chen Z Zhang S . LA-D-B1, a novel abemaciclib derivative, exerts anti-breast cancer effects through CDK4/6. (2024) 48:847–60. doi: 10.32604/biocell.2024.050868

[117] Zhang X Ge X Jiang T Yang R Li S . Research progress on immunotherapy in triple‑negative breast cancer (Review). Int J Oncol. (2022) 61:95. doi: 10.3892/ijo.2022.5385 35762339 PMC9256074

[118] Peng P Qiang X Li G Li L Ni S Yu Q . Tinengotinib (TT-00420), a novel spectrum-selective small-molecule kinase inhibitor, is highly active against triple-negative breast cancer. Mol Cancer Ther. (2023) 22:205–14. doi: 10.1158/1535-7163.mct-22-0012 36223547 PMC9890131

[119] Alečković M Li Z Zhou N Qiu X Lulseged B Foidart P . Combination therapies to improve the efficacy of immunotherapy in triple-negative breast cancer. Mol Cancer Ther. (2023) 22:1304–18. doi: 10.1201/9780429263545-3 PMC10618734 37676980

[120] Telli ML Litton JK Beck JT Jones JM Andersen J Mina LA . Neoadjuvant talazoparib in patients with germline BRCA1/2 mutation-positive, early-stage triple-negative breast cancer: exploration of tumor BRCA mutational status. Breast Cancer. (2024) 31:886–97. doi: 10.1007/s12282-024-01603-4 38869771 PMC11341741

[121] Tutt ANJ Garber JE Kaufman B Viale G Fumagalli D Rastogi P . Adjuvant olaparib for patients with BRCA1- or BRCA2-mutated breast cancer. N Engl J Med. (2021) 384:2394–405. doi: 10.1007/s12282-023-01451-8 34081848 PMC9126186

[122] Pantelidou C Jadhav H Kothari A Liu R Wulf GM Guerriero JL . STING agonism enhances anti-tumor immune responses and therapeutic efficacy of PARP inhibition in BRCA-associated breast cancer. NPJ Breast Cancer. (2022) 8:102. doi: 10.1038/s41523-022-00471-5 36068244 PMC9448789

[123] Marcut L Brata RD Barb AC Manole A Stef DG Dumitru CS . Targeting DNA damage response and immune crosstalk in cancer: Mechanistic insights and therapeutic opportunities. Int J Mol Sci. (2025) 26:11271. doi: 10.3390/ijms262311271 41373427 PMC12692409

[124] Dixon-Douglas J Loibl S Denkert C Telli M Loi S . Integrating immunotherapy into the treatment landscape for patients with triple-negative breast cancer. Am Soc Clin Oncol Educ Book. (2022) 42:1–13. doi: 10.1200/edbk_351186 35649211

[125] Dong C Wu J Chen Y Nie J Chen C . Activation of PI3K/AKT/mTOR pathway causes drug resistance in breast cancer. Front Pharmacol. (2021) 12:628690. doi: 10.3389/fphar.2021.628690 33790792 PMC8005514

[126] Cretella D Ravelli A Fumarola C La Monica S Digiacomo G Cavazzoni A . The anti-tumor efficacy of CDK4/6 inhibition is enhanced by the combination with PI3K/AKT/mTOR inhibitors through impairment of glucose metabolism in TNBC cells. J Exp Clin Cancer Res. (2018) 37:72. doi: 10.1186/s13046-018-0741-3 29587820 PMC5872523

[127] Hassan A Aubel C . The PI3K/Akt/mTOR signaling pathway in triple-negative breast cancer: A resistance pathway and a prime target for targeted therapies. Cancers (Basel). (2025) 17:2232. doi: 10.3390/cancers17132232 40647529 PMC12248877

[128] Bullock KK Richmond A . Beyond anti-PD-1/PD-L1: Improving immune checkpoint inhibitor responses in triple-negative breast cancer. Cancers (Basel). (2024) 16:2189. doi: 10.3390/cancers16122189 38927895 PMC11201651

[129] Zhu S Wu Y Song B Yi M Yan Y Mei Q . Recent advances in targeted strategies for triple-negative breast cancer. J Hematol Oncol. (2023) 16:100. doi: 10.1186/s13045-023-01497-3 37641116 PMC10464091

[130] Li G Hu J Cho C Cui J Li A Ren P . Everolimus combined with PD-1 blockade inhibits progression of triple-negative breast cancer. Cell Signal. (2023) 109:110729. doi: 10.1016/j.cellsig.2023.110729 37257766

[131] Rodriguez BL Gibbons DL . MEK inhibition invigorates chemoimmunotherapy by tumor mitophagy-induced CXCL10 expression. Cell Rep Med. (2022) 3:100506. doi: 10.1016/j.xcrm.2021.100506 35106515 PMC8784763

[132] Franklin DA Sharick JT Ericsson-Gonzalez PI Sanchez V Dean PT Opalenik SR . MEK activation modulates glycolysis and supports suppressive myeloid cells in TNBC. JCI Insight. (2020) 5:e134290. doi: 10.1172/jci.insight.134290 32634121 PMC7455066

[133] Shuai Y-Y Wang P-P Zhang H-J Ao H Peng W Zhang H . ROS regulation by natural products: A promising therapeutic approach for breast cancer. (2025) 49:2299–333. doi: 10.32604/biocell.2025.071569

[134] Cheng TC Sayseng JO Tu SH Juan TC Fang CL Liao YC . Curcumin-induced antitumor effects on triple-negative breast cancer patient-derived xenograft tumor mice through inhibiting salt-induced kinase-3 protein. J Food Drug Anal. (2021) 29:622–37. doi: 10.38212/2224-6614.3387 35649138 PMC9931023

[135] Han X Zhao N Zhu W Wang J Liu B Teng Y . Resveratrol attenuates TNBC lung metastasis by down-regulating PD-1 expression on pulmonary T cells and converting macrophages to M1 phenotype in a murine tumor model. Cell Immunol. (2021) 368:104423. doi: 10.1016/j.cellimm.2021.104423 34399171

[136] Huang L Xue R Zhu M Xu S Luo Y Qin C . Triptolide's impact on ACER1 signaling: Inducing autophagy for triple-negative breast cancer suppression. Pathol Res Pract. (2025) 266:155823. doi: 10.1016/j.prp.2025.155823 39879681

[137] Yang Z Zhang Q Yu L Zhu J Cao Y Gao X . The signaling pathways and targets of traditional Chinese medicine and natural medicine in triple-negative breast cancer. J Ethnopharmacol. (2021) 264:113249. doi: 10.1016/j.jep.2020.113249 32810619

[138] Deng Z Chen G Shi Y Lin Y Ou J Zhu H . Curcumin and its nano-formulations: Defining triple-negative breast cancer targets through network pharmacology, molecular docking, and experimental verification. Front Pharmacol. (2022) 13:920514. doi: 10.3389/fphar.2022.920514 36003508 PMC9393234

[139] Zhang J Zheng J Chen H Li X Ye C Zhang F . Curcumin targeting NF-κB/Ubiquitin-Proteasome-System axis ameliorates muscle atrophy in triple-negative breast cancer cachexia mice. Mediators Inflammation. (2022) 2022:2567150. doi: 10.1155/2022/2567150 35132306 PMC8817892

[140] Cheng T Wang C Lu Q Cao Y Yu W Li W . Metformin inhibits the tumor-promoting effect of low-dose resveratrol, and enhances the anti-tumor activity of high-dose resveratrol by increasing its reducibility in triple negative breast cancer. Free Radic Biol Med. (2022) 180:108–20. doi: 10.1016/j.freeradbiomed.2022.01.010 35038549

[141] Giri P Camarillo IG Sundararajan R . Enhancement of reactive oxygen species production in triple negative breast cancer cells treated with electric pulses and resveratrol. Explor Target Antitumor Ther. (2023) 4:42–56. doi: 10.37349/etat.2023.00122 36937321 PMC10017187

[142] Chen Y Yang J Wang C Wang T Zeng Y Li X . Aptamer-functionalized triptolide with release controllability as a promising targeted therapy against triple-negative breast cancer. J Exp Clin Cancer Res. (2024) 43:207. doi: 10.1186/s13046-024-03133-5 39054545 PMC11270970

[143] Li ZX Zang L Li YQ Zhang M Zhao DM Han XY . Anti-cancer research progress of triptolide: Mechanisms of action, structural modifications, nanodelivery systems and clinical challenges. Drug Des Devel Ther. (2026) 20:601715. doi: 10.2147/dddt.s601715 42221354 PMC13222053

[144] Zhu M Liu Y Wen Z Tan H Li S Yu X . Exploration of traditional Chinese medicine comprehensive treatment of triple negative breast cancer based on molecular pathological mechanism. Breast Cancer (Dove Med Press). (2025) 17:289–304. doi: 10.2147/bctt.s511059 40236879 PMC11998019

[145] Schipilliti FM Drittone D Mazzuca F La Forgia D Guven DC Rizzo A . Datopotamab deruxtecan: A novel antibody drug conjugate for triple-negative breast cancer. Heliyon. (2024) 10:e28385. doi: 10.1016/j.heliyon.2024.e28385 38560142 PMC10981107

[146] Tolaney SM de Azambuja E Kalinsky K Loi S Kim SB Yam C . Sacituzumab govitecan plus pembrolizumab for advanced triple-negative breast cancer. N Engl J Med. (2026) 394:354–66. doi: 10.1056/nejmoa2508959 41564397

[147] Morrison L Okines A . Systemic therapy for metastatic triple negative breast cancer: Current treatments and future directions. Cancers (Basel). (2023) 15:3801. doi: 10.3390/cancers15153801 37568617 PMC10417818

[148] Schreiber AR O'Bryant CL Kabos P Diamond JR . The emergence of targeted therapy for HER2-low triple-negative breast cancer: a review of fam-trastuzumab deruxtecan. Expert Rev Anticancer Ther. (2023) 23:1061–9. doi: 10.1080/14737140.2023.2257885 37742278

[149] Bardia A Hurvitz SA Tolaney SM Loirat D Punie K Oliveira M . Sacituzumab govitecan in metastatic triple-negative breast cancer. N Engl J Med. (2021) 384:1529–41. doi: 10.1056/nejmoa2028485 33882206

[150] Oh KS Nam AR Bang JH Jeong Y Choo SY Kim HJ . Immunomodulatory effects of trastuzumab deruxtecan through the cGAS-STING pathway in gastric cancer cells. Cell Commun Signal. (2024) 22:518. doi: 10.1186/s12964-024-01893-3 39449023 PMC11515331

[151] Forveille S Leduc M Sauvat A Kroemer G Kepp O . Datopotamab deruxtecan induces hallmarks of immunogenic cell death. Cell Stress. (2025) 9:194–200. doi: 10.15698/cst2025.08.311 40800161 PMC12342961

[152] Koh SA . Immunotherapy in triple-negative breast cancer: mechanisms of resistance and emerging approaches: a narrative review. J Yeungnam Med Sci. (2026) 43:17. doi: 10.12701/jyms.2026.43.17 41645627 PMC12962768

